# Permanent pacemaker implantation complicated by anchoring sleeve embolization through cephalic vein: case report and discussion of management strategies

**DOI:** 10.1093/ehjcr/ytaf416

**Published:** 2025-08-29

**Authors:** Saleh Altaf, Thivanka Witharana, Alexander Tindale, David Gareth Jones

**Affiliations:** Cardiology Department, Harefield Hospital, Guy’s and St Thomas’ NHS Foundation Trust, London, UK; Cardiology Department, Harefield Hospital, Guy’s and St Thomas’ NHS Foundation Trust, London, UK; Cardiology Department, Harefield Hospital, Guy’s and St Thomas’ NHS Foundation Trust, London, UK; Cardiology Department, Harefield Hospital, Guy’s and St Thomas’ NHS Foundation Trust, London, UK

**Keywords:** Sleeve embolization, Pacemaker complication, Pulmonary embolism, Case report

## Abstract

**Background:**

Anchoring sleeve embolization is a rare complication of first-time permanent pacemaker (PPM) implantation, and there is little guidance on how to manage such an eventuality.

**Case summary:**

An 82-year-old lady underwent PPM implantation for symptomatic 9-second sinus pause. During procedure, the anchoring sleeve from the atrial lead embolized through cephalic vein to the left superior lingular artery. The patient remained haemodynamically stable and multimodality imaging demonstrated only a small affected area of lung with patent pulmonary blood flow distal to the sleeve. Therefore, the patient was managed with anticoagulation alone and has remained well at 26-month follow-up.

**Discussion:**

Most case reports dealing with embolization of pacing apparatus to the pulmonary vasculature document endovascular retrieval. We lay down a framework of considerations for assessing management strategies to help guide practitioners to the most efficacious treatment plan. This includes factors affecting the risks of leaving the foreign body in place and outlines the rationale for no active treatment, through anticoagulation, and towards endovascular and surgical retrieval.

Learning points

- Pacing sleeve embolization through cephalic vein is a rare complication during pacemaker implantation.
- In case of anchoring sleeve embolization into the vein, the pacing lead should not be removed as it can lead to distal embolization into pulmonary vasculature.
- In the absence of haemodynamic compromise and low risk of infection, leaving the embolized sleeve in pulmonary vasculature can be an effective strategy.

## Introduction

Permanent pacemaker (PPM) implantation is a routine procedure with relatively low complication rate of 4%–5%, commonly lead displacement, pneumothorax, bleeding, and infection.^1,2^ Migration of lead fragments occurs mainly during explanation and complicate 0.06% of procedures.^3^ Around 2% of these rare embolization events involve the anchoring sleeve of the pacemaker lead.^4^ Sleeve embolization during implantation is exceedingly rare and has been reported once through subclavian vein and, to the best of our knowledge, never through cephalic vein.^5^

We present the case of a patient who suffered distal embolization of the anchoring sleeve of atrial pacing lead during her first PPM implantation through cephalic vein. Due to rarity, there is little consensus on the best strategy to manage such cases, whether by conservative management, anticoagulation alone, or retrieval. We discuss the case and the rationale for conservative management in our patient before proposing a framework of considerations to guide management in cases of pacing apparatus embolization.

## Summary figure

**Figure:**
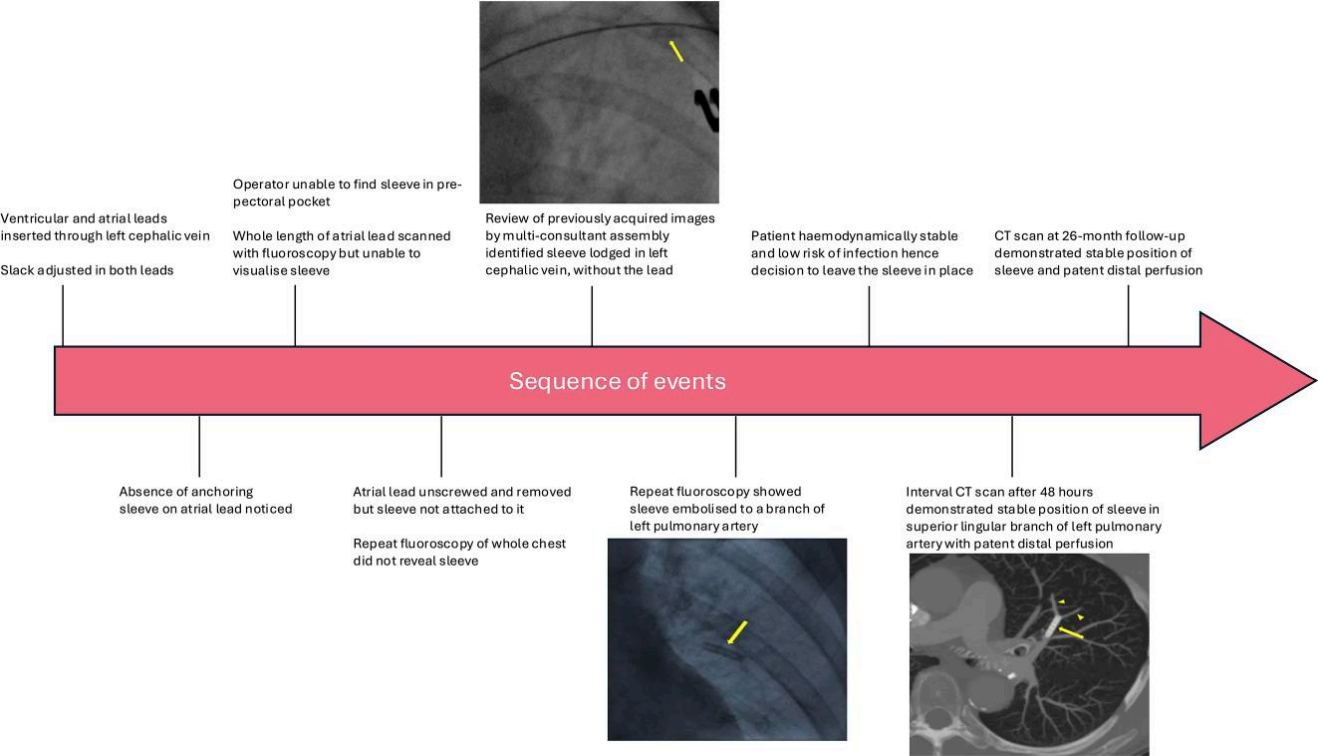


## Case presentation

An 82-year-old woman was admitted following a Stokes–Adams attack 8 days after ablation for atrial fibrillation (AF). She had a background of hypothyroidism, transient ischaemic attack (TIA), and paroxysmal AF. She was anticoagulated with apixaban 5 mg twice daily due to her age, female sex, and a previous TIA. She had good biventricular function and was taking bisoprolol 2.5 mg once daily.

On admission, she had a heart rate of 80 beats/minute and a blood pressure of 107/61 mmHg. However, her electrocardiogram showed sinus rhythm, first degree heart block with a PR interval of 274 ms compared to 247 ms post-ablation. A further 9-second pause on the coronary care unit, despite normal biochemistry results and cessation of bisoprolol, led to an urgent permanent pacemaker insertion.

A left infraclavicular incision was made, and a pre-pectoral pocket was created. Left cephalic vein was used to deliver active atrial and ventricular leads (both St Jude Medical Tendril STS, ABBOTT MEDICAL UK LIMITED) to the right atrial appendage and right ventricular septum, respectively. The slack was adjusted in both leads, and cephalic vein was tied off.

At this juncture, it was noted that the anchoring sleeve on the atrial lead was missing. The operator tried to find the sleeve in the pre-pectoral pocket but could not locate it. The length of the lead was screened with fluoroscopy, but the sleeve was not visualized. The lead was then unscrewed and removed but there was no sleeve attached to it. The whole chest was screened with fluoroscopy, but sleeve was not visible.

**Figure 1:**
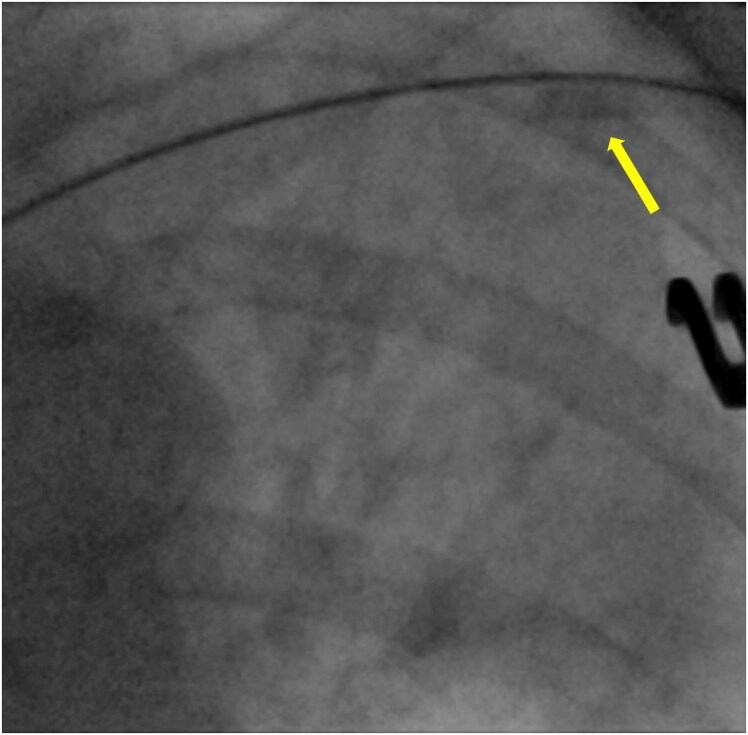
Fluoroscopic image showing pacing sleeve (yellow arrow) in left cephalic vein without atrial lead passing through it.

**Figure 2:**
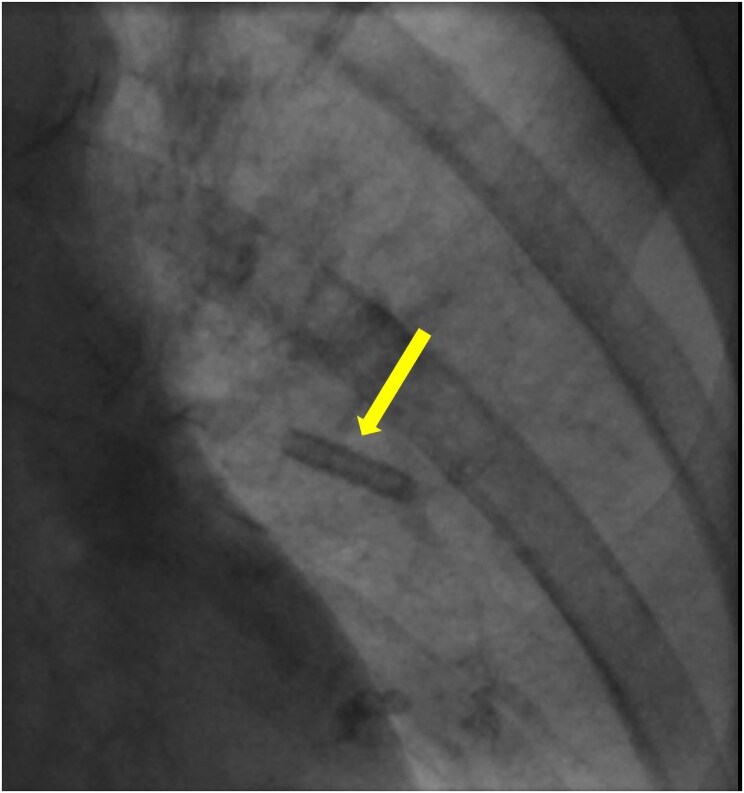
Right anterior oblique view on fluoroscopy showing pacing sleeve (yellow arrow) lodged in a branch of left pulmonary artery.

A multi-consultant meeting was assembled and review of previously acquired images showed an isolated sleeve without the lead lodged in left cephalic vein (*Figure 1*). Repeat fluoroscopy in multiple views did not show the sleeve in cephalic vein rather the sleeve appeared to have embolized to a branch of left pulmonary artery (*Figure 2*). Plausible mechanism of entry of sleeve into the cephalic vein could be that it was inadvertently pushed on the naked (without sheath) atrial lead during ventricular lead slack adjustment. Embolization further into the venous system and pulmonary artery occurred after the lead was removed.

The patient was asymptomatic and haemodynamically stable. Therefore, it was thought that the risks associated with retrieval outweighed the risks of leaving it in place. A new atrial lead was implanted via ultrasound-guided axillary puncture. The remainder of the procedure was uncomplicated, the leads were connected to a ST Jude Assurity MRI generator (ABBOTT MEDICAL UK LIMITED) and the pocket closed.

**Figure 3:**
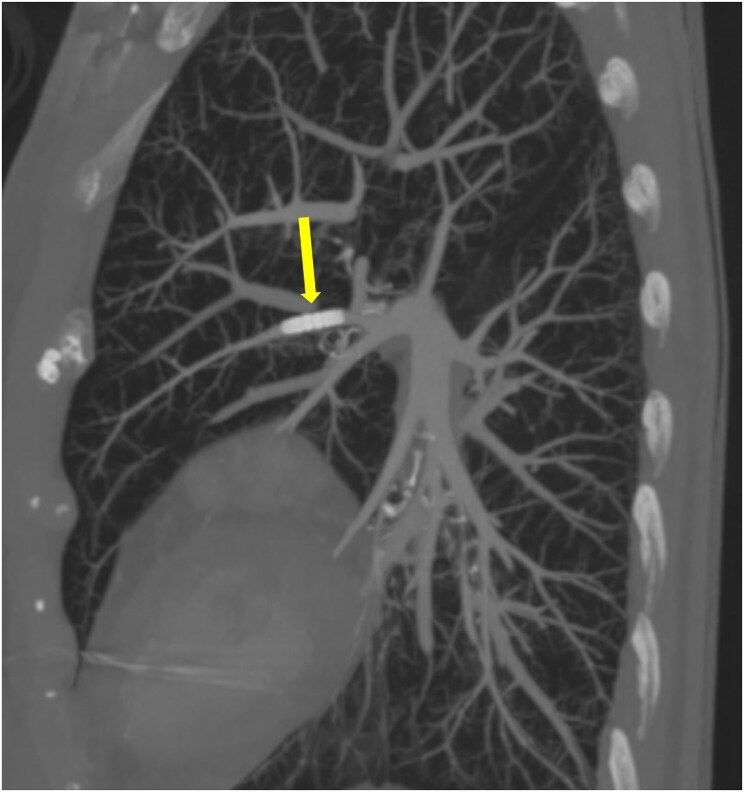
DECTPA sagittal view showing anchoring sleeve (yellow arrow) in superior lingular branch of left pulmonary artery.

**Figure 4:**
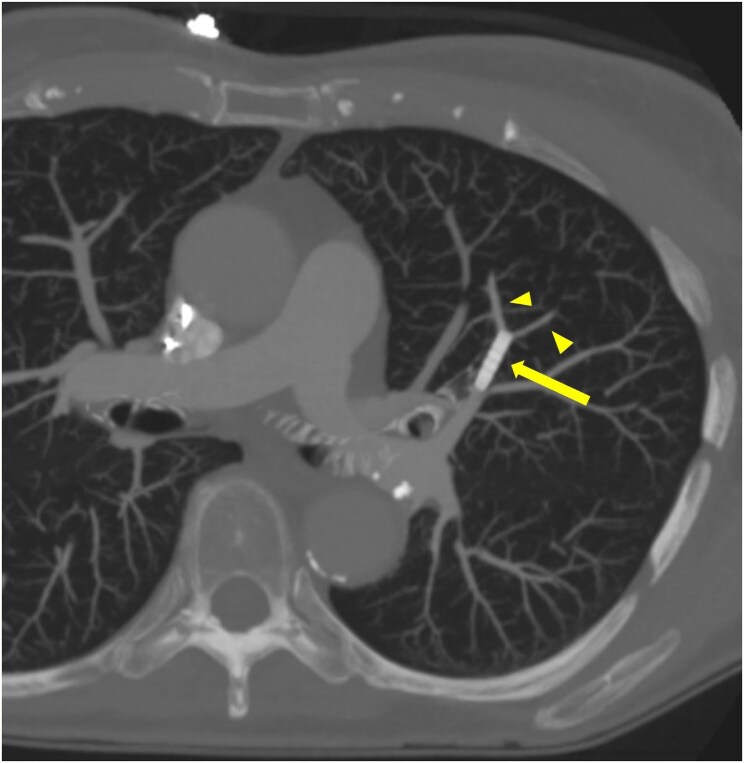
DECTPA cross-sectional view showing anchoring sleeve in superior lingular artery (yellow arrow). Perfusion can be seen after bifurcation (yellow arrowheads).

A dual-energy computed tomography angiogram of the pulmonary arteries (DECTPA) was performed 48 h later. It demonstrated that the sleeve was lodged in the superior lingular branch of left pulmonary artery, its position was stable, and it was supplying a small area of lung parenchyma. There was patent flow and no perfusion defect distal to it (*Figures 3* and *4*).

The patient was discharged on apixaban 5 mg twice daily for both AF and thrombus formation prevention around the sleeve, prophylactic anti-biotics and bisoprolol 2.5 mg once daily.

**Figure 5:**
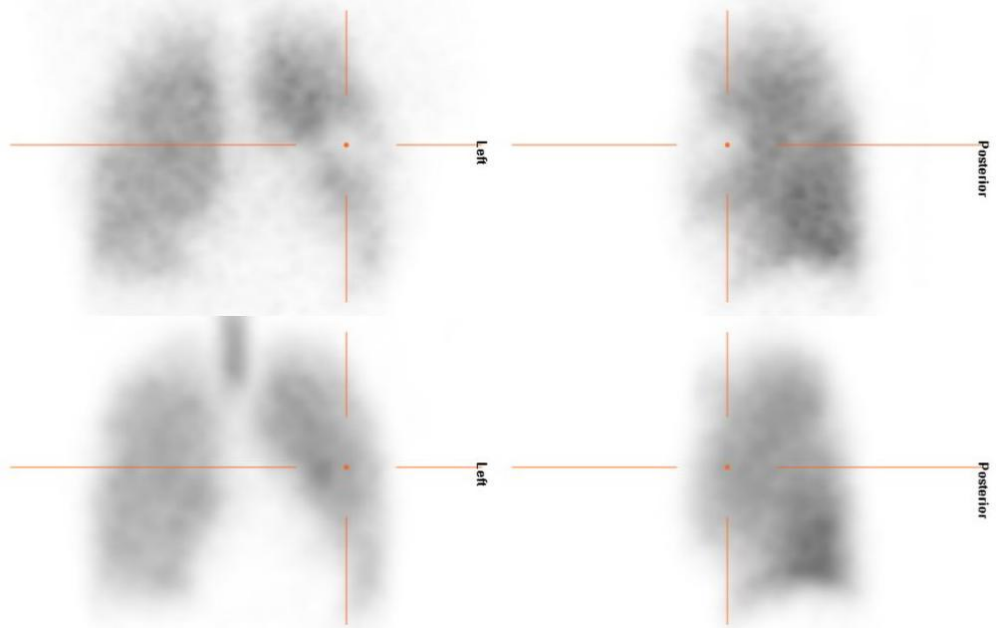
Ventilation–perfusion scan showing a small mismatch in left middle zone.

Two weeks after discharge patient underwent a ventilation–perfusion scan using single positron emission computed tomography (SPECT-CT) which showed reduced perfusion, as expected due to narrowing of arterial lumen by sleeve, and preserved ventilation in the left middle zone in keeping with the location of the sleeve (*Figure 5*). The diminutive size of perfusion defect reinforced the team’s belief in continuing anticoagulation only.

Since then, our patient has remained clinically well and asymptomatic at 26 months and a subsequent DECTPA has showed stability of the anchoring sleeve with patent distal flow.

## Discussion

The embolization of parts of pacing leads including anchoring sleeve is relatively well-described in the literature in cases of pacemaker extraction and is estimated to complicate up to 0.06% of procedures.^3^ In contrast, the migration of pacing sleeves during pacemaker implantation is much rarer. In fact, there is a paucity of data on the frequency with which these sleeves embolize. Pacing apparatus embolization usually directs the fragments towards the pulmonary arterial tree: This phenomenon has been documented for almost 50 years.^6^

When pacing sleeves are missing, there are four main management strategies described in the literature:

1. Conservative management: If the embolized object remains in pulmonary vasculature, the location and haemodynamic effects of the culprit embolus can dictate the risks and benefit of conservative management. For example, the more distal the embolus is in the pulmonary vasculature, the smaller the benefit of removal and the higher the technical difficulty and risk of removal.^7^
2. Lead implantation: If the sleeve is missing from the proximal site where it is to be sutured to the pre-pectoral fascia, the safest strategy may be to not remove the lead and implant it as it is. This way, there is little chance of the sleeve embolizing further into the pulmonary arterial tree. This strategy has been used in a similar situation.^8^
3. Endovascular retrieval: This is relatively safe and well-tolerated and has been described in case reports and series. A single-centre experience of 45 retrievals had a success rate above 90%. The only reported anchoring sleeve removal in this study occurred after the sleeve was embolized during device extraction for infected endocarditis.^9^
4. Surgical retrieval: If endovascular retrieval is technically challenging.

To choose the correct course of action, the risks of leaving the suture sleeve in place should be considered. These could be summarized mainly as:

- Infection of the foreign body: This will be most relevant if there is a high risk of infection during the procedure, for example if lead fragment embolization were to occur during a lead extraction for infective endocarditis.^10^
- Pulmonary embolus causing significant haemodynamic collapse. If a proximal embolus occurs it can provoke haemodynamic collapse or severe hypoxia.^11^
- Symptoms including pain caused by pleuritis. In the absence of large pulmonary infarction this is usually a late complication.^12^

The balance between passive and active management should guide treatment strategy. To use this case as an example: there were no symptoms or haemodynamic effects and no obvious pulmonary infarct. Furthermore, the pacing sleeve was sterile and this was a first-time implantation, thus the risk of infection was low. Therefore, we decided to leave the anchoring sleave in place.

A more difficult decision needs to be made regarding anticoagulation. In this case, the patient required anticoagulation for AF but without an underlying anticoagulation indication a judgement call must be made. In one case report, the decision to anti-coagulate was driven by a relatively large infarct visible on post-procedure plain chest radiograph.^13^ In a contrasting report, a patient was initially discharged without anticoagulation and subsequently returned after two years with a pulmonary embolus around the foreign object at which point anticoagulation was started.^11^ A pragmatic, albeit evidence-free, approach may be to treat with six months of oral anticoagulation, as one may for an unprovoked pulmonary embolism, to allow time for endothelialization of the pacing sleeve.^7^

## Data Availability

Non-identifiable data underlying this article will be made available upon reasonable request to the corresponding author.

## Lead author biography

The lead author graduated from the University of Health Sciences, Pakistan and is currently working as a clinical fellow in cardiology at Harefield Hospital, London.
